# A Card

**Published:** 1892-01

**Authors:** E. Van Goidtsnoven

**Affiliations:** Atlanta, Ga.


### A CARD.

ATLANTA, GA., NOV. 22, 1891.

*Editors Atlanta Medical and Surgical Journal:*

Our acknowledgments and thanks are due the gentleman who complimented us with an editorial allusion in the last number of the *Southern Medical Record.* The insinuation is, that we have accepted a pittance at the hands of the East Tennessee, Virginia and Georgia Railway, and our competency is questioned, if not assailed.

With becoming modesty, we hold that we are as honorable and as ethical in our methods as the writer of that criticism.

The blame, the fault, is not with the physician who accepts the place, but with the profession which deals in double weights and measures, smiles on some of its members and frowns on others.

Why does not the medical profession, through its national, State and local organizations, condemn and anathematize both the offer and the acceptance ?

Why does not the Georgia Medical Association repudiate its former president, vice-president and censor who wrote the tariff for the Georgia Railroad, a tariff similar to and almost identical with that of the East Tennessee, Virginia and Georgia Railroad ?

Nine-tenths of the medical profession of Atlanta pity Olmsted for refusing Ford's bill of fare and approve of Cooper accepting it! Again, nine-tenths of the medical profession of Atlanta pity Olmsted for rejecting Ross' proffered fee bill and approve of Nicolson accepting it! Consistency, thou art a jewel!

We of the East Tennessee, Virginia and Georgia Railroad are held in ridicule and almost contempt, for having subscribed to Drake's fee bill.

To use a French expression, our Code of Ethics "n'a ni cul ni tete." We would like to find its head, and know something of tail. We would like to know why it is *censorially* ethical to propose and ridiculously little to accept ? We would like to know why the Georgia Railroad and the Richmond and Danville

Railroad are spared the criticism of that editorial in the *Southern Medical Record*?

Throw off your mask and confirm our suspicions! We suspect that Nemesis had more to do with the inspiration of that editorial, than a wounded sense of the violation of ethical rules and methods.

With respect to the insinuation of "poor pay, poor surgeons," we have every reason to flatter ourselves that we are as competent as the denouncer.

E. VAN GOIDTSNOVEN, M. D.

---

A MARTYR FOR SUBSTITUTION.—In a recent issue of the *National Advertiser* the following item was copied from the *Bulletin of Pharmacy*:

M. Herrouet, a pharmacist of Mans, France, has retired to the seclusion of one of the French prisons for three months, the better to consider the evils of substitution. A physician had prescribed a hypodermatic injection of apomorphine, for a child three months old. The prescription was presented to M. Herrouet for dispensing, who, having no apomorphine, substituted morphine hydrochlorate, under the impression, as he admits, that the result would be the same. The physician, ignorant of the change, administered the injection; the child immediately fell into a comatose condition and died of poisoning shortly afterwards. M. Herrouet was prosecuted for homicide by imprudence, and the court decided that three months' solitary and careful thought would probably convince him that it is always best to dispense a prescription as written or not dispense it at all. We would just remark *sotto voce* that substitution on physicians' prescriptions is not confined to France.

